# Synthesis and Evaluation of Poly(Sodium 2-Acrylamido-2-Methylpropane Sulfonate-co-Styrene)/Magnetite Nanoparticle Composites as Corrosion Inhibitors for Steel

**DOI:** 10.3390/molecules19021713

**Published:** 2014-01-30

**Authors:** Gamal A. El-Mahdy, Ayman M. Atta, Hamad A. Al-Lohedan

**Affiliations:** 1 Chemistry Department, College of Science, King Saud University, Riyadh 11451, Saudi Arabia; 2 Chemistry Department, Faculty of Science, Helwan University, Helwan 11795, Egypt; 3 Petroleum Application Department, Egyptian Petroleum Research Institute, Cairo 11727, Egypt

**Keywords:** magnetite, nanoparticle, nanocomposite, sodium 2-acrylamido-2-methylpropane sulfonate-co-styrene, corrosion inhibitor

## Abstract

Self-stabilized magnetic polymeric composite nanoparticles of coated poly-(sodium 2-acrylamido-2-methylpropane sulfonate-co-styrene)/magnetite (PAMPS-Na-co-St/Fe_3_O_4_) were prepared by emulsifier-free miniemulsion polymerization using styrene (St) as a monomer, 2-acrylamido-2-methylpropane sulfonic acid sodium salt (AMPS-Na) as an ionic comonomer, *N,N*-methylenebisacrylamide (MBA) as crosslinker, hexadecane (HD) as a hydrophobic solvent, and 2,2-azodiisobutyronitrile (AIBN) as an initiator in the presence of hydrophobic oleic acid coated magnetite particles. Hydrophobic oleic acid coated magnetite particles with an average size of about 7-10 nm were prepared with the new modified water-based magnetite ferrofluid, synthesized by a chemical modified coprecipitation method. The morphology and the particle size distributions of the crosslinked PAMPS-Na-co-St/Fe_3_O_4_ composite were observed and analyzed by transmission electron microscopy (TEM). The average Fe_3_O_4_ content of PAMPS-Na-co-St/Fe_3_O_4_ was determined by thermogravimetric analysis (TGA). The inhibitory action of PAMPS-Na-co-St/Fe_3_O_4_ towards steel corrosion in 1 M HCl solutions has been investigated by polarization and electrochemical impedance spectroscopy (EIS) methods. Polarization measurements indicate that PAMPS-Na-co-St/Fe_3_O_4_ acts as a mixed type-inhibitor and the inhibition efficiency increases with inhibitor concentration. The results of potentiodynamic polarization and EIS measurements clearly showed that the inhibition mechanism involves blocking of the steel surface by inhibitor molecules via adsorption.

## 1. Introduction

Steel has become an important part of daily operations in the petroleum and gas pipeline industries. Hydrochloric acid (HCl) is widely used in industry for oil-well acidizing, acid descaling of boilers, acid cleaning, pickling, cooling towers, and heat exchangers to remove deposits and other corrosion products. In the past few years, many efforts have been focused on the fabrication of metal oxide nanoparticles as corrosion inhibitors due to their unique chemical and physical properties [1,2,3,4]. One of the most effective routes to protect low-carbon steels against corrosion consists in the formation of self-organized oxide coatings to provide additional protection against corrosion. TiO_2_ [5,6,7,8,9], SiO_2_ [10] and ZrO_2_ [11] particles have been incorporated into polymer matrices to improve the corrosion resistance of the coatings. Nanosized fillers have attracted great attention in industrial applications, due to the superior properties they exhibit, even at low concentrations, such as strength, mechanical stability and barrier film compactness. Among the many materials used for this purpose, iron oxides, besides being cheap, seem to satisfy the requirements for anticorrosive protection; an example of these pigments is magnetite and magnetite coatings (MC) play an important role in corrosion protection. [11]. However, when the material is either exposed to a high humidity atmosphere or aqueous media, or subjected to the effects of aggressive ions, the protection provided by MC coatings may become insufficient, and additional action must be taken to inhibit the corrosion. In this context it was reported that the interaction of magnetite as a core with polymer shell composities increases their stability to environment [11]. The passivation of MC coatings formed on low-carbon steel surfaces allows improvement of their protective characteristics. It is also important that anticorrosive materials should be environmentally acceptable, as this is considered one of the biggest concerns that producers of such materials have at the moment. 

The potentiodynamic polarization behavior suggested a positive effect of the fillers on the corrosion resistance provided by organic coatings, although no information on the mechanism was provided. Montoya *et al.* [12] electrosynthesized polypyrrole-magnetite/silane (PPy-Fe_3_O_4_/GPTMS-TEOS-γ-APS) coatings and the anticorrosive properties of the PPy-Fe_3_O_4_/GPTMS-TEOS-γ-APS bilayer coating applied on carbon and stainless steel substrates were assessed by open circuit potential (OCP), electrochemical impedance spectroscopy (EIS) and Raman spectroscopy. The electrochemical characterization has shown that the anodic polarization of the substrate by the conductive polymer was beneficial for the carbon steel substrate, whereas it induced pitting corrosion in stainless steel. It was also demonstrated that the addition of Fe_3_O_4_ to the PPy matrix slows down its reduction rate increasing its stability and that the presence of a barrier top-coat (like a silane film) is fundamental to preserve the effective anticorrosion performance of the PPy-Fe_3_O_4_ composite coating during long term exposures.

Magnetite is one of the most promising nanoparticles employed in anticorrosion coatings [13,14]. To date, many methods have been developed to prepare Fe_3_O_4_ nanoparticles, including coprecipitation [15], microwave thermal-hydrolysis [16], oxidation of Fe(OH)_2_ by H_2_O_2_ [17], micron-scale capsules [18], and thermal decomposition of Fe(CO)_5_ [19]. Among them, solution coprecipitation has been proved to be one of the most powerful and practical approaches in the synthesis of Fe_3_O_4_ nanoparticles. Using this method, Fe_3_O_4_ nanoparticles of various shapes (such as cubic, pyramidal and tetrahedral) have been achieved [20]. The difficulties in the synthesis of well-dispersed and active Fe_3_O_4_ nanoparticles may be attributed to the adsorption of surfactant molecules on the surface of nanoparticles leading to agglomeration and a propensity to oxidation of the nanoparticles.

To enhance the compatibility between the magnetic Fe_3_O_4_ nanoparticles and water, and to control and/or tailor of the surface properties of the nanoparticles, surface modification is a necessity for magnetic Fe_3_O_4 _nanoparticles. The surfaces of nanoparticles are covered by a capping shell, which inhibits the agglomeration and oxidation of nanoparticles and enhances the application of MC as an anticorrosive coating [21,22]. Ferromagnetic magnetite (Fe_3_O_4_) with a cross-linked polymer nanogel is considered the most interesting among the magnetic nanogels. In addition, ferromagnetic magnetite exhibits strong magnetic properties, superparamagnetic behavior and good barrier properties toward corrosion processes. There are several parameters that affect the magnetic properties (superparamagnetism and ferromagnetism) of magnetite such as particle diameter, particle shape and particle aggregation [20]. Nanogel particles have advantages over normal organic inhibitors due to the formation of uniform thin films on the surface of steel, which entirely cover all the surface without any defects. Regulators were proposed to play an important role in the arrangement of surfactant molecules on the surface of nanoparticles, which is quite intriguing. In the present work, it was thought valuable to investigate the application of magnetite coated poly(sodium 2-acrylamido-2-methylpropane sulfonate-co-styrene)/magnetite nanocomposite (PAMPS-Na-co-St/Fe_3_O_4_) as a corrosion inhibitor for steel in an aggressive acidic medium (1 M HCl) using potentiodynamic polarization curves and electrochemical impedance spectroscopy (EIS). As far as we know, it is the first time the protective properties of these new compounds based on Fe_3_O_4_ nanoparticles dispersed in a composite have been investigated. The interaction between the poly(sodium methacrylate) structures and their arrangement over the Fe_3_O_4_ surface are discussed.

## 2. Results and Discussion

**Scheme 1:**
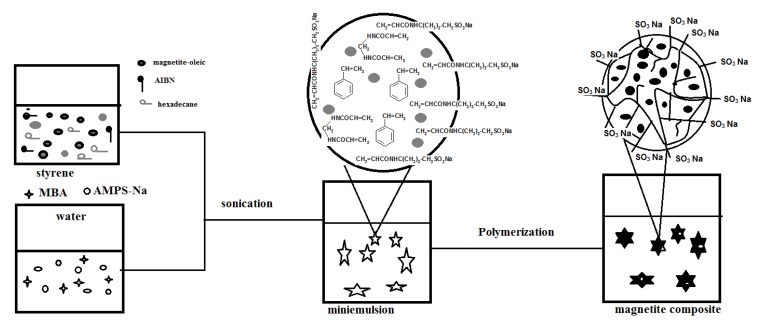
Synthesis of PAMPS-Na-co-St/magnetite composite.

Nanogel composites containing dispersed magnetite nanoparticles can be prepared magnetite nanoparticles in styrene and HD solution. In this way, the miniemulsion polymerization process will take place in the styrene (St) monomer droplets containing the magnetite particles. The hydrophobic magnetite coated nanoparticles were prepared according to a modified previous method [22] based on the cheapest and most environmentally friendly co-precipitation method, which involves the simultaneous precipitation of Fe^2+^ and Fe^3+^ ions after reaction of KI with FeCl_3_. The iodine was separated during the reaction by precipitation and the oleic acid-coated magnetite was produced after precipitation in basic aqueous media in the presence of oleic acid as stabilizer. The transformation of the hydrophilic-hydrophobic character of magnetite particles is fundamental for encapsulating them successfully inside the polymer particles. Moreover, the hydrophilicity of the magnetite nanoparticle surface may affect their compatibility and dispersability with the hydrophilic poly(sodium 2-acrylamido-2-methylpropane sulfonate-co-styrene). This may lead to the encapsulation of inorganic particles inside or on the surface layer of the latex particles. The proposed procedure for preparing poly(sodium 2-acrylamido-2-methylpropane sulfonate-co-styrene)/magnetite nanocomposites by miniemulsion polymerization in the absence of an emulsifier using hexadecane (HD) as hydrophobe and AMPS-Na as stabilizer is illustrated in Scheme 1.

As can be seen, AMPS-Na, MBA were dissolved in DDI water, and AIBN was dissolved in the mixture of HD and oleic Fe_3_O_4_-St. The two solutions were then mixed together under stirring and sonicated. A stable minimulsion was obtained, the temperature was then raised to 70 °C and monomer droplets polymerized. It is expected that the sulfonic groups of AMPS-Na anchored covalently onto the particle surface avoiding the migration that occurs with conventional emulsifier molecules, thus enhancing the stability of the particles and increasing the hydrophilicity of the copolymer with respect to the encapsulated oleic acid-coated magnetite particles [23]. Moreover, it is expected that the concentration of magnetite particles is favored to increase in the core of the composite particles.

### 2.1. Characterization of PAMPS-Na-co-St/Magnetite Composite

**Figure 1:**
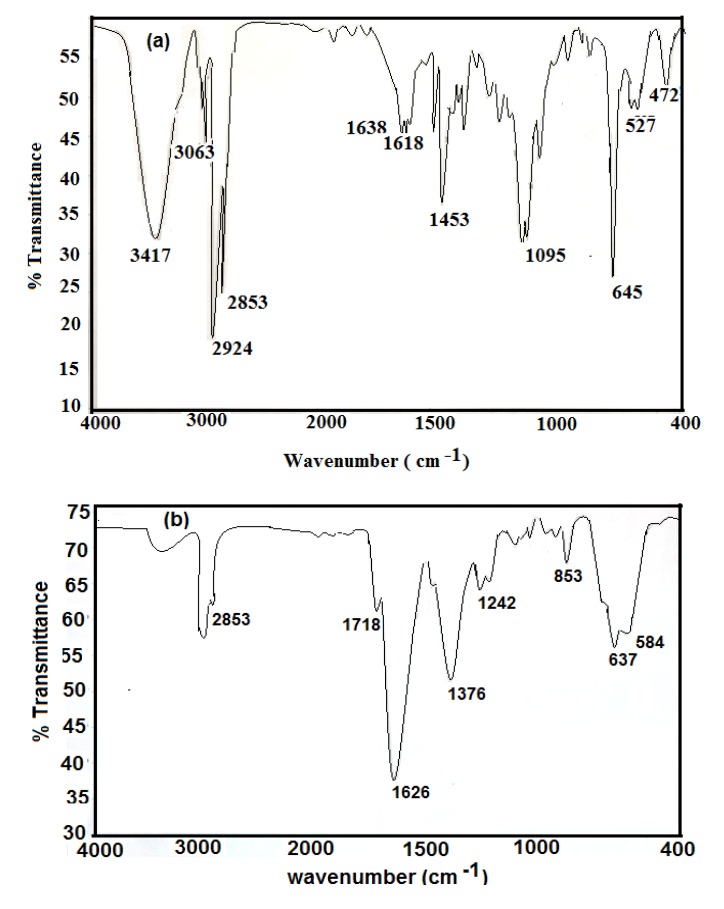
FTIR specta of (**a**) oleic acid coated magnetite and (**b**) PAMPS-Na-co-St/magnetite composite.

FTIR analysis was used to study the chemical structures of the prepared nanoparticles. The IR spectra of hydrophobic oleic Fe_3_O_4_ and PAMPS-Na-co-St/Fe_3_O_4_AA-Na magnetite particles are presented in Figure 1a,b, respectively.

It was observed that the IR spectra of all samples clearly reveal the presence of strong IR absorption bands between 400 and 700 cm^−1^, which are the characteristic absorption peaks of the Fe-O vibrations related to Fe_3_O_4_. Magnetite formation can be confirmed through the presence of the 584 and 637 cm^−1^ bands assigned to the stretching and torsional vibration modes of the magnetite. In both magnetite coated phases bands are seen at higher frequency in the near IR region than previously reported, where the characteristic absorption band for Fe-O in bulk Fe_3_O_4_ appeared at 570 and 375 cm^−1^[24].

However, in the present case the Fe-O band shift towards higher wavenumbers (584 and 637 cm^−1^) which may be due to the breakage of a large number of bonds of surface atoms, resulting in rearrangement of localized electrons on the particle surface and the surface bond force constant then increases as Fe_3_O_4_ is reduced to nanoscale dimensions, so that the absorption bands shift to higher wavenumbers [24]. In Figure 1a Fe_3_O_4_ nanoparticles coated with oleic acid show characteristic bands of asymmetric and symmetric COO- stretching (1453 and 1638 cm^−1^) [25], along with a band at 1050 cm^‒1^ characteristic of a C-O single bond which demonstrates that oleic acid was chemisorbed onto the nanoparticles as a carboxylate. Fe-O stretching of magnetite was also observed at 578 and 628 cm^−1^. The strong band at 3424 cm^−1^ is attributed to the symmetric O-H stretching vibration of hydroxyl groups that are absorbed onto that part of the particle surface not occupied by surfactant groups as well as ν (O–H) for H-bonded OH groups (from oleic acid). Two sharp bands at 2924 and 2854 cm^−1^ are attributed to the asymmetric and symmetric CH_2_ stretches, respectively. The oleic acid surfactant molecules in the adsorbed state on magnetite are subjected to the solid surface field. As a result, the characteristic oleic acid bands are shifted to a lower frequency region, indicating that the hydrocarbon chains in the monolayer surrounding the iron nanoparticles are in a closed pack crystalline state [25]. Figure 1b shows the characteristic C=O band (present at 1712 cm^−1^ for pure oleic acid and AMPS-Na). The appearance of a band at 3350 cm^−1^ (NH group of MBA) in Figure 1b indicates the crosslinking of AMPS-Na with the MBA crosslinker. The appearance of a band at 850 cm^−1^, corresponding to the C-H out of plane bending of the phenyl group indicates the incorporation of styrene into the chemical structure of the magnetite polymer composite.

**Figure 2:**
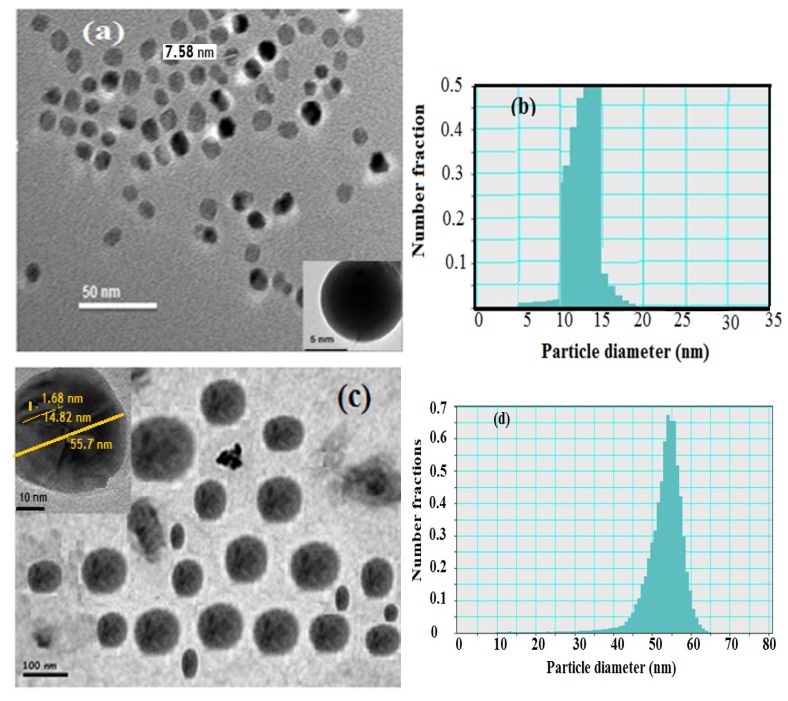
TEM micrographs and histograms of (**a**) and (**b**) oleic coated magnetite, (**c**) and (**d**) PAMPS-Na-co-St/magnetite composite.

Transmission electron microscopy (TEM) was used to characterize the hydrophobic oleic Fe_3_O_4_ and PAMPS-Na-co-St/Fe_3_O_4_AA-Na magnetite particle nanocomposites as illustrated in Figure 2. The hydrophobic magnetite stabilized with oleic acid nanoparticles analyzed by TEM showed a spherical shape with a narrow size distribution (Figure 2a,b).

The image reveals the formation of monodispersed nanoparticles with an average size of 8–13 nm confirming that the proposed scheme is an effective method for the preparation of monodisperse magnetic nanoparticles. Number—(dn^−^), weight—(dw^−^), and volume-average (dv^−^) diameters as well as polydispersity indices (PDI) were calculated from the PSDs [26]. The data of dn^−^, dw^−^, dv^−^ and PDI of the hydrophobic oleic Fe_3_O_4 _are 10.7, 11.8, 13.3 nm and 1.05, respectively.

The particle size distributions appear to be quite narrow and the particle shape is reasonably spherical. The presence of magnetite in the PAMPS-Na-co-St polymeric matrix was identified by the black dots over the grey dots, as shown in Figure 2b. Another feature present in Figure 2b, and in many of the other TEM images of the polymer PAMPS-Na-co-St/magnetite coated nanoparticles, is that the particles are not in contact with their nearest neighbors. During preparation of the TEM sample, solvent-dispersed particles with swollen shells may be deposited in a close-packed array on the carbon support. The absence of pure polymer particles and free magnetite particles in Figure 2b indicated that monomer droplet nucleation was achieved entirely by using an emulsifier-free miniemulsion polymerization technique. The dn^−^, dw^−^, dv^−^ and PDI values are 67, 73, 79 nm and 1.1, respectively, indicating the formation of almost monodisperse PAMPS-Na-co-St/magnetite composite. In addition, in the TEM micrographs (Figure 2b), the existence of a few large composite particles can be observed in the PAMPS-Na-co-St/magnetite latex.

**Figure 3:**
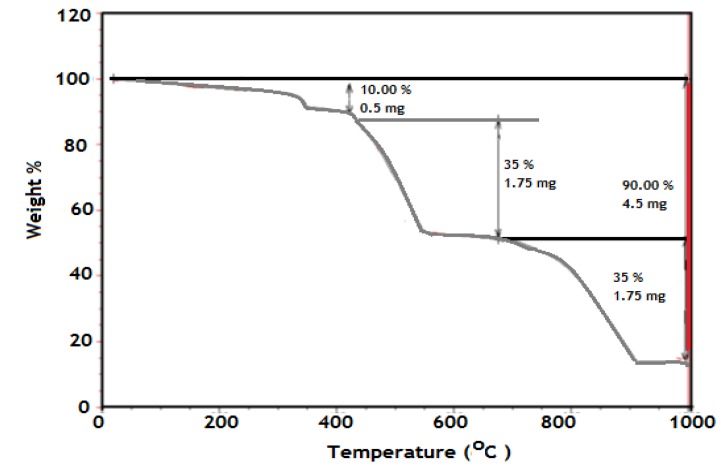
TGA thermogram of PAMPS-Na-co-St/magnetite composite.

It is well established that the TGA is an effective analysis tool to determine the inorganic contents in polymer composites. The polymer should decompose completely when the temperature is high enough, but if there are inorganic materials in the sample, they should remain and can be measured. The TGA curves of PAMPS-Na-co-St/magnetite composite are presented in Figure 3.

The thermogram exhibits a multiple-stage thermal decomposition. The first stage is in the 25–143 °C range and corresponds to the loss of moisture present in the sample (2%). The initial decomposition temperature (Ti) of the nanocomposite was 279.9 °C. This peak is attributed to the thermal decomposition of the amide side-groups of AMPS-Na. The weight loss in the range 220–320 °C corresponds to the temperature of the transformation from magnetite to hematite. This transformation appears as a multi-step process, which is composed of the gradual transformation of the magnetite nanoparticles to maghemite (γ-Fe_2_O_3_). The weight loss (5.4%) in the 297.2–318.9 °C range is attributed to the transformation from maghemite to hematite (α-Fe_2_O_3_). These two thermal events are consistent with the previously reported phase-transformation temperature range of magnetite. This is followed by weight loss within the 300–470 °C temperature range, which is attributed to the thermal decomposition of the crosslinker. This result confirms that the incorporation of a magnetic nanogel is helpful for the improvement of the thermal stability of the nanocomposites. As can be seen in Figure 3, when the temperature reached about 850 °C the weight of the sample was constant and the residue was 9.295% of the original sample mass. Since there was no increase in weight resulting from the oxidation of Fe_3_O_4_ to Fe_2_O_3_ because the TGA experiment was carried out under a nitrogen atmosphere, this fact indicated that the Fe_3_O_4_ content in the PAMPS-Na-co-St/magnetite latex was approximately 10%.

### 2.2. Surface Activity of PAMPS-Na-co-St/Magnetite Composite

**Figure 4:**
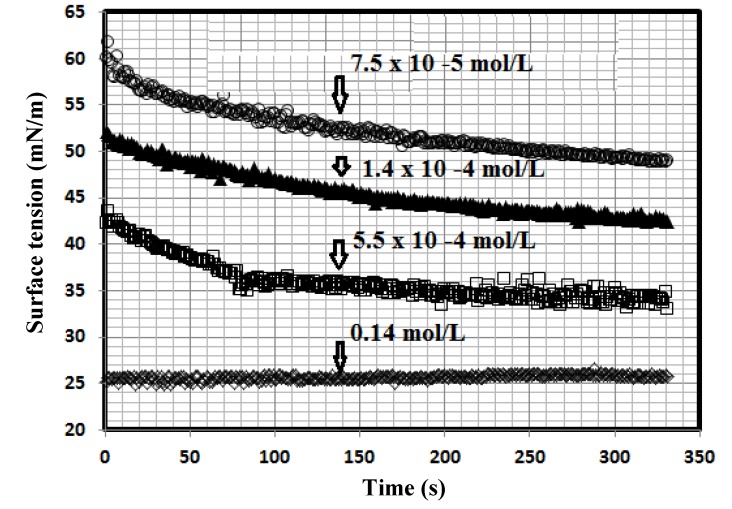
Surface tension ageing time relationship of PAMPS-Na-co-St/magnetite composites of different concentrations in 1 M HCl at 25 °C.

A literature survey on the topic of surface activity of dispersed nanoparticles showed little research about this subject. In previous works [27,28,29], the determination of the surface activities of the nanogels and correlation with their size, morphology and chemical structures was the research target. In this respect, measuring the surface activity of PAMPS-Na-co-St/magnetite composite in both aqueous and 1 M HCl is a goal of the present work to measure the ability of particles to be dispersed in aqueous media or adsorb at the water/air interface. In the present article, it was expected that the prepared PAMPS-Na-co-St/magnetite composite would possess surface activity due to the presence of both the hydrophilic moieties of AMPS (amide and sulfonate) and hydrophobic moieties (phenyl, butane, oleic and polymer backbone groups) in the chemical structure of the crosslinked composite nanoparticles. In this respect, the dynamic surface tension for different concentrations of the prepared PAMPS-Na-co-St/magnetite composite was measured at the water/air interface for aqueous solution and 1 M HCl at a temperature of 25 °C. The relation between the surface tension of different concentrations of dispersed PAMPS-Na-co-St/magnetite and time of measurements in 1 M HCl was selected for representative samples and is illustrated in Figure 4.

The data presented in Figure 4 indicate that the dispersed PAMPS-Na-co-St/magnetite reached its surface tension equilibrium after time intervals ranging from 5 to 15 min. Moreover, the equilibrium time was reduced with increasing PAMPS-Na-co-St/magnetite concentration and replacement of water with 1 M HCl. These data indicated that the prepared surfactants are strongly adsorbed at the interfaces in 1 M HCl solutions. On the other hand, it was observed that the surface tension data of the aqueous nonionic surfactant solutions was not affected by 1 M HCl.

It is well known that the interactions between amphiphilic particles and solvents affect the micellization, aggregation and adsorption of particles and they are based on the critical aggregation concentrations (cac), which were determined by the surface balance method. The cac data of PAMPS-Na-co-St/magnetite were determined from the abrupt changes of the plotted data of surface tension (γ) *versus* the solute concentration (lnC) in 1 M HCl and aqueous solution at 25 °C and are listed in Table 1.

**Table 1:** Surface activity data of PAMPS-Na-co-St/magnetite composite in water and 1 M aqueous HCl solution.

Medium	cac mol/L	γcac mN/m	Δγ mN/m	(−∂γ/∂lnc)T	Гmax × 10 ^1^^0^ mol/ cm^2^	A_min_ nm^2^/molecule
Water	0.001	25.3	46.8	5.5	2.22	0.075
1 M HCl	0.0005	25.2	46.9	8.9	3.58	0.046

The cmc data indicated that the PAMPS-Na-co-St/magnetite forms aggregates in 1 M HCl at lower concentration, and more so than in aqueous solution. This can be attributed to the conversion of SO_3_Na groups to SO_3_H groups and the increased hydrophobicity of magnetite particles in HCl solutions [30].

The adsorption effectiveness of the prepared PAMPS-Na-co-St/magnetite expressed by the maximum reduction of surface tension which was calculated from the equation, Δγ = γ_water_ − γ_cac_, the concentration of the prepared surfactants at the solvent-air interface, Г_max_, and the area per molecule at the interface, A_min_, were calculated and are listed in Table 1 too. The surface excess concentration of the prepared PAMPS-Na-co-St/magnetite at the interface can be calculated from the surface or interfacial tension data using the following equation [31]: Г_max_ = 1/RT × (−∂γ/∂lnc)T, where (−∂γ/∂lnc)T is the slope of the plot of γ *versus* ln c at constant temperature (T), and R is the gas constant (in J mol^−1^ K ^−1^). The Г_max_ values were used for calculating the minimum area (A_min_) at the aqueous–air interface. The area per molecule at the interface provides information about the degree of packing and the orientation of the adsorbed surfactants, when compared with the dimensions of the molecules obtained from models. From the surface excess concentration, the area per molecule at the interface is calculated using the equation: A_min_ = 10^16^/N Гmax, where N is Avogadro’s number. The data listed in Table 1 indicated that the cmc values were reduced from 0.001 to 0.0005 mol/L in aqueous 1 M HCl, which can be attributed to the lower dispersion of PAMPS-Na-co-St/magnetite in acid solution at high PAMPS-Na-co-St/magnetite concentration. It was previously concluded that decreasing the cmc values indicated a high tendency of surfactants to adsorb at the liquid interface [32]. Also, the area occupied at the interface is increased [33]. These data indicated that the prepared PAMPS-Na-co-St/magnetite favors micellization in bulk 1 M HCl solution compared to aqueous solution, which may reflect its greater tendency to adsorb at the metal/liquid interface more than the air/water interface. It was also suggested that the spherical PAMPS-Na-co-St/magnetite particles are deformed at the water/air interface and become lenslike, causing more efficient adsorption [34,35].

### 2.3. Stability PAMPS-Na-co-St/Magnetite to HCl

**Figure 5:**
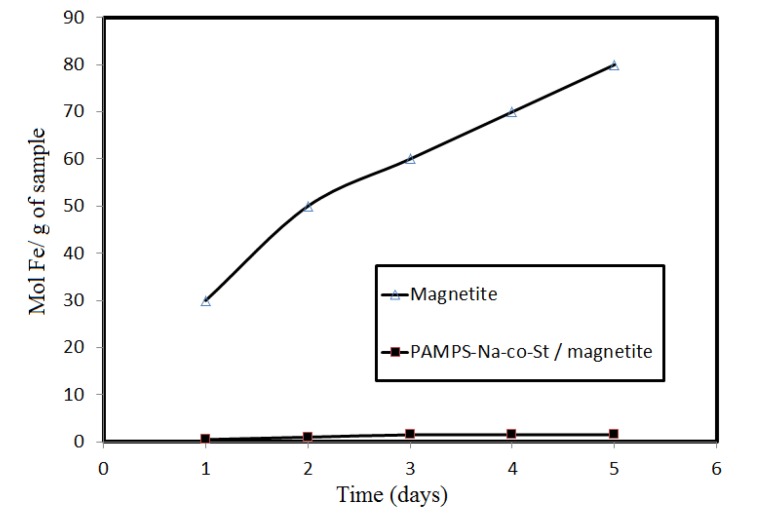
The amount of Fe dissolved in 1 M HCl within 5 days from Fe_3_O_4_ and PAMPS-Na-co-St/magnetite composite.

The production of magnetite nanoparticles (MNPs) with high stability and biocompatibility presents one of the greatest challenges. To address this, MNPs should be covered with a quite inert external shell, in order to protect the magnetic core against chemical changes. Uncoated magnetic nanoparticles are highly susceptible to oxidation when exposed to the atmosphere and are also susceptible to leaching under acidic conditions [36]. In addition to convenient magnetic properties and low toxicity and price, Fe_3_O_4_ exhibits high surface to volume ratios, depending on the particle size, which is associated to its ability to undergo surface chemical modifications that can enhance the capacity for heavy metal adsorption in water treatment processes. Inorganic polymers, such as silica, have been used as stabilizing agents for iron oxide and the silica coating has attractive properties, including high biocompatibility [37], adsorption capacity, acid-base properties, insolubility in most solvents, and chemical and thermal stability. Besides enlarging the application scope of the material, one objective of coating magnetite is to improve its chemical stability. In this work, we evaluated the effect of PAMPS-Na-co-St coating on the chemical stability of magnetite toward HCl acid. A sample of magnetite was mixed with a solution of HCl for a certain period and the dissolved Fe(II) and Fe(III) were analyzed by atomic absorbance spectroscopy. Figure 5 shows that PAMPS-Na-co-St/magnetite is more stable than the examined magnetite nanoparticles toward HCl.

This can be attributed to the protection afforded by sulfonate propyl groups from PAMPS and the phenyl groups of styrene, which have no tendency to interact with the protons of HCl. The formed coating is able to protect magnetite from acid. It can be seen that magnetite coated with oleic acid tends to have lower stability than that with core-shell PAMPS-Na-co-St/magnetite composite. This might be attributed to the fact oleic acid has lower stability in 1 M HCl due to the interaction of the COOH groups and the double bonds of oleic acid with the HCl protons [2], affecting the oleic acid layer coated on magnetite. The decrease in the acid stability of magnetite could be attributed to a decrease in the surface area of the magnetite after removal of the oleic acid coat.

### 2.4. Polarization Measurements

**Figure 6:**
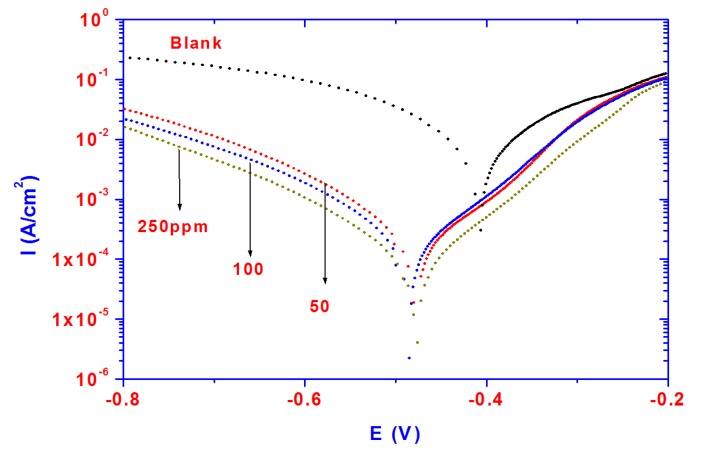
Polarization curves for steel in 1 M HCl solution containing different PAMPS-Na-co-St/magnetite concentrations.

The dissolution of steel in acidic solutions is a major problem in industrial applications due to aggressiveness of acid, which causes the degradation of materials exposed to acidic media. Steel is widely exposed to the acidic environments used in oil well acidizing, acid cleaning and acid pickling. Potentiodynamic polarization plots for steel specimens in 1 M HCl solution in the absence and presence of different concentrations (50–250 ppm) of PAMPS-Na-co-St/magnetite particles are shown in Figure 6. It is evident from the data presented in Figure 6 that the cathodic current density decreased with the addition of inhibitor to the blank solution and shifts towards more negative values as the inhibitor concentration increases.

The results can be accounted for by the reduction of the cathodic process. In addition, the anodic current density decreases with the addition of inhibitor to the blank solution due to the formation of an inhibitive layer which reduces the anodic dissolution of steel. It can be concluded that the presence of PAMPS-Na-co-St/magnetite particles results in a marked shift in the cathodic and anodic branches of the polarization curves due to blocking of the active sites [38,39]. The cathodic reaction was strongly retarded, with an initial E_CORR_ displacement in the negative direction, and the anodic reaction was also retarded strongly inhibited. Increasing the inhibitor concentration to 250 ppm favored the inhibitory action. The decrease in the current densities of both anodic and cathodic reactions suggests that the inhibitor can be considered a mixed type inhibitor. The inhibition efficiency IE% was calculated using the following equation:

IE% = 1 − i_corr(1)_/i_corr(2)_ × 100 (1)

where i_corr(1)_ and i_corr(2)_ are the corrosion current densities of steel in the presence and absence of inhibitor, respectively. The electrochemical parameters associated with corrosion process as corrosion current density (i_corr_), corrosion potential (E_corr_), cathodic Tafel slope (B_c_), anodic Tafel slope (B_a_), and inhibition efficiency (IE%)] are given in Table 2.

**Table 2:** Inhibition efficiency of PAMPS-Na-co-St/magnetite composite values for steel in 1 M HCl with different concentrations of inhibitor calculated by the polarization and EIS methods.

Polarization Method						EIS Method		
	B_a_ (mV)	B_c_ (mV)	E_corr_ (V)	i_corr_ mA/cm^2^	IE%		R_ct_ Ohm	IE%
Blank	147.0	141.00	−0.4034	7.45	-		1.80	-
50 ppm	101.4	104.4	−0.4777	0.186	97.50		578	99.5
100	99.9	112.9	−0.4848	0.177	97.62		649	99.6
250	89.1	105.1	−0.4768	0.074	99.00		693	99.7

The data of Table 2 also shows how the addition of PAMPS-Na-co-St/magnetite particles decreases the corrosion current density. The inhibitive action of nanoparticles may be attributed to the adsorption and formation of a protective film on the electrode surface. The barrier film formed on the steel surface reduces both the anodic and cathodic reactions, which results in the decrease in both current densities and confirming that these nanoparticles act as mixed type corrosion inhibitors. The data presented in Table 2 reveals that the values of B_a_ and B_c_ for inhibited solutions were lower than for uninhibited ones. The corrosion potentials (E_corr_) are shifted to more negative values in the presence of nanoparticles, indicating that the inhibitor had a stronger influence on the cathodic reduction reaction. The cathodic shifts of (E_corr_) and the decreases of the corresponding current densities (i_corr_) with increasing inhibitor concentration can be attributed to a higher number of adsorbed nanoparticles on the steel surface, which entirely cover the steel surface and decrease the corrosion rate.

### 2.5. Electrochemical Impedance Spectroscopy (EIS)

**Figure 7:**
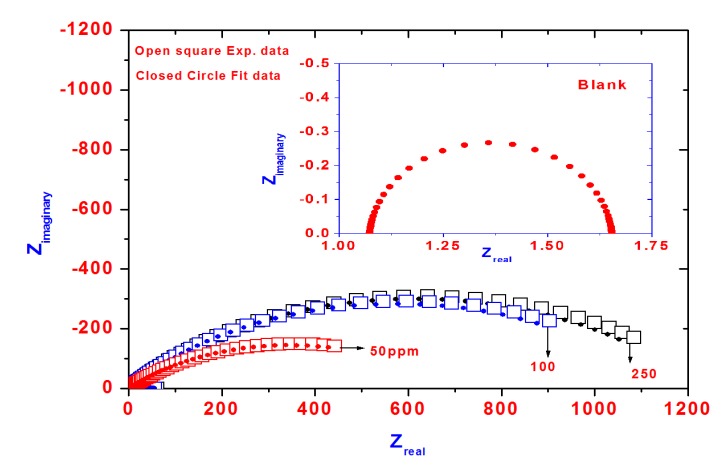
Nyquist diagram for steel in 1 M HCl solution containing different PAMPS-Na-co-St/magnetite concentrations showing experimental and fit data.

The effects of the inhibitor concentration on the impedance behavior of aluminum in 1.0 M HCl solution have been studied and the corresponding Nyquist plots are given in Figure 7. The insert represents the Nyquist plot of blank solution.

The impedance spectra exhibit one single capacitive loop, which indicates that the corrosion reaction is controlled by a charge transfer reaction. It is worth noting that the presence of inhibitor does not change the mechanism of steel dissolution [40]. It is evident that the impedance diagrams are not perfect semicircles, which is related to the frequency dispersion as a result of the roughness and inhomogeneity of the steel surface [41].

**Figure 8:**
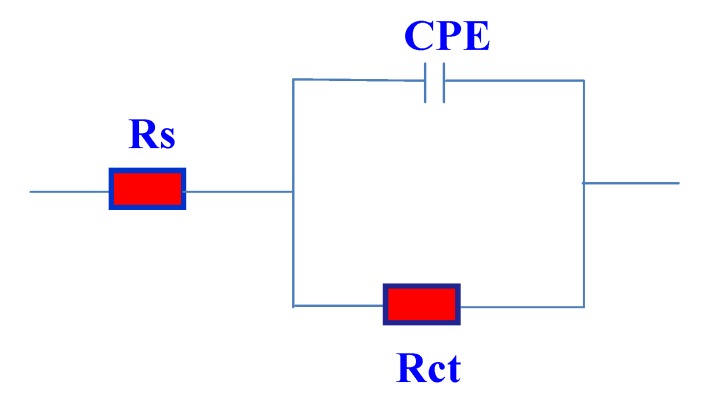
Equivalent circuit used for fitting the impedance data.

The equivalent circuit model used to fit the experimental results is shown in Figure 8. In this equivalent circuit, Rs is the solution resistance, Q is a constant phase element (CPE) and Rct is the charge transfer resistance. The impedance of CPE is described as [42]:

Z_CPE_ = 1/Q(jω)^n^ (2)

where Q is the pseudo capacitance, j is the imaginary function (√−1), ω is the angular frequency and n corresponds to the deviation from the ideal behavior of a pure capacitor. The inhibition efficiency (IE%) at different inhibitor concentrations were calculated by using the following equation [43]:

IE% = 1 − Rct_(1)_/Rct_(2)_ × 100 (3)

where Rct_(1)_ and Rct_(2)_ are the charge transfer resistances in the HCl solution in the absence and in the presence of the inhibitors, respectively. The analysis of data (Table 2) shows that the charge transfer resistance and the IE values increased with as the inhibitor concentration increased. The results can be explained on the basis of an enhancement of the adsorption process of inhibitor molecules on the steel surface with increasing inhibitor concentration, which resulted in larger surface coverage and an increase in IE%. The thickness of this protective layer increases with an increase in inhibitor concentration because more inhibitor will be adsorbed on the steel surface, leading to an increase in the IE%. As shown from Figure 2 there is no aggregation of the nanoparticles. Formation of aggregates will decrease the surface area of the magnetic nanoparticles, thereby limiting and undermining the effectiveness of magnetic nanoparticles to protect the steel surface. It is expected that the interactions between the nanoparticles will limit the mobility of the particles to be adsorbed on the steel surface and decrease the efficiency. In addition, the higher efficiency may be attributed to fast adsorption kinetics and higher number of the adsorbed nanoparticle particles on the steel surface as the nanoparticles concentration increases. The potentiodynamic polarization and EIS measurement results clearly showed that the inhibition mechanism involves blocking of the steel surface by inhibitor molecules via adsorption. The results obtained from the EIS measurements are in good agreement with those obtained from the polarization method.

## 3. Experimental

### 3.1. Chemicals

Anhydrous ferric chloride, potassium iodide and aqueous (25%) ammonium hydroxide solution used as reagents for preparation of magnetite were purchased from Aldrich Chemical Co. (St. Louis, MO, USA). Oleic acid (OA), 2-acrylamido-2-methylpropane sulfonic acid sodium salt (AMPS-Na), poly(vinyl pyrrolidone) (PVP) with molecular weight 40,000 g/mol, *N,N*-azobisisobutyronitrile (ABIN), *N,N*′-dimethylformamide, acetone and hexadecane, were obtained from Sigma Chemicals (St. Louis, MO, USA). All reagents were used as received, and double deionized (DDI) water prepared with a UVO ultrapurification system (Millipore, Billerica, MA, USA) was used throughout the work.

### 3.2. Preparation of Hydrophobic Magnetite Nanoparticles

The hydrophobic magnetite nanoparticles were made by coating magnetite with oleic acid. In this respect, a solution of anhydrous FeCl_3_ (40 g) in distilled water (300 mL) containing PVP (3 g) was prepared and named aqueous solution A. Further, potassium iodide (13.2 g, 0.08 mol) was dissolved in distilled water (50 mL) to prepare an aqueous solution B. The aqueous solutions A and B are then mixed together at room temperature, stirred and allowed to reach equilibrium for one hour while bubbling with pure N_2_ to keep the mixture oxygen free throughout the preparation procedure. Oleic acid (3 mL) diluted in ethanol (100 mL) was added after heating the reaction mixture at 50 °C under vigorous stirring, then NH_4_OH (25%, 200 mL) was added dropwise to the above solution. After that, the reaction mixture was stirred for another 0.5 h at 70 °C. Finally, the oleic acid-stabilized Fe_3_O_4_ nanoparticles were separated from the mixture by ultracentrifugation, washed with water three times and with ethanol two times. The hydrophobic magnetite nanoparticles were re-suspended in hexadecane at 5% (w/v) concentration.

### 3.3. Preparation of Poly(Sodium 2-Acrylamido-2-methyl Propane Sulfonate-co-Styrene)/Magnetite Nanocomposite

Poly(sodium 2-acrylamido-2-methyl propane sulfonate-co-styrene)/magnetite nanocomposite (PAMPS-Na-co-St/Fe_3_O_4_) can be prepared using emulsifier-free miniemulsion polymerization [25]. In this respect, the reactants recipe has the following composition: solution A: hexadecane (HD, 29.3 g) containing magnetite nanoparticle solution (0.5 g), AIBN (0.1 g) and styrene (2 g). Solution B contains MBA (0.1 g), and 65 wt % DDI water containing AMPS-Na (3 g). The polymerization solutions A and B were pre-emulsified. AMPS-Na monomer and MBA crosslinker was dissolved in DDI water and AIBN was dissolved in the mixture of HD and Fe_3_O_4_-St. The two solutions were mixed together under stirring provided by a magnetic bar stirrer for 20 min and miniemulsified (Model 450 sonifier, Branson, Danbury, CT, USA) in an ice-cooled bath under a nitrogen atmosphere. The miniemulsified optimum conditions are: output control, 8 kHz duty cycle, 80%; sonication time, 10 min. The emulsifier-free miniemulsion polymerization was conducted in 250 mL glass bottle immersed in a thermostatic bath. The bottle was tumbled end-over-end at 49 rpm. The bottle was purged with nitrogen for 10 min after miniemulsification and the reaction temperature was kept at 60 °C for 24 h in the bath. The polymerization yields a brownish inverse latex containing poly(sodium 2-acrylamido-2-methyl propane sulfonate-co-styrene)/magnetite nanocomposite. The particles were isolated by distilling most of hexadecane and water off under vacuum and then drying in an oven at 40 °C for 24 h. The yield of the produced composite was 84%, calculated after redispersion in ethanol three times and reprecipitation using a ultracentrifuge at 120,000 rpm, followed by air drying at 40 °C.

### 3.4. Characterization

Fourier Transform infrared (FTIR) spectroscopic analysis of the samples was performed using a Spectrum One FTIR spectrometer (Perkin-Bhaskar-Elmer Co., Boston, MA, USA). The morphology and structure of the prepared magnetic nanoparticles were determined using high-resolution transmission electron microscopy (HR-TEM). HR-TEM images of the nanocomposites were recorded using a JEM-2100 F (JEOL, Tokyo, Japan) at an acceleration voltage of 150 kV. The particle size distributions (PSDs) were determined from TEM microphotographs on representative samples of more than 1,000 particles of magnetic latexes and analyzed using the Bolero software (AQ Systems, Sweden).

Thermogravimetric analysis (TGA, Universal V3.5B TA Instruments, (TGA, Universal V3.5B TA Instruments, New Castle, DE, USA) was used to determine the average Fe_3_O_4_ content of representative poly(sodium 2-acrylamido-2-methyl propane sulfonate-co-styrene)/magnetite nanocomposite sample. The Fe_3_O_4_ content was given according to the weight percentage of the residue remaining after thermal analysis from room temperature to 1,000 °C under a nitrogen atmosphere.

The surface tension measurements of the prepared poly(sodium acrylate)/magnetite nanocomposites in 1 M HCl aqueous solution were carried out at different molar concentrations at a temperature of 25 °C by means of the pendant drop technique using a drop shape analyzer model DSA-100 (Kruss, Hamburg, Germany).

The resulting product (0.5 g) was mixed with HCl solution (1 M, 15 mL), shaken for 5 min and kept at room temperature. From the mixture, 1 mL of the substrate was collected at one day intervals for 5 days, and the content of dissolved iron was analyzed with atomic absorption spectroscopy following the direct aspiration into an air-acetylene flame using a Perkin-Elmer 2380 atomic absorption instrument (Norwalk, CT, USA).

### 3.5. Electrochemical Measurements

The steel electrode was made of steel rod with a surface area of 0.785 cm^2^. It has the following chemical composition (wt %): 0.14% C, 0.57% Mn, 0.21% P, 0.15% S, 0.37% Si, 0.06% V, 0.03% Ni, 0.03% Cr and the remainder Fe. Polarization measurements were carried out in a conventional three electrode cell consisting of steel as the working electrode (WE), a saturated calomel electrode as reference electrode (RE) and a platinum sheet as counter electrode (CE). The electrode surface was abraded, just before experiments, by different grades of emery paper and then rinsed abundantly with deionized water. The electrochemical and impedance measurements were carried out with a Solartron 1470E system (potentiostat/galvanostat) with a Solartron 1455A frequency response analyzer (Cambridge, UK). The electrochemical impedance spectra were measured at 10 mV AC amplitude over a frequency range of 0.01 Hz–10 kHz. Collection and evaluation of experimental data was accomplished by the Multistate software. The impedance data were analyzed and fitted with the simulation ZView 3.3c, equivalent circuit software.

## 4. Conclusions

Emulsifier-free miniemulsion polymerization using an ionic monomer (AMPS-Na) was used to prepare self-stabilized magnetic polymeric composite particles. The results showed that the encapsulation of magnetite using this technique was successful. AMPS-Na works as a high-efficiency stabilizer in the preparation of PAMPS-Na-co-St/magnetite by emulsifier-free miniemulsion polymerization. The distribution of magnetite particles inside the PAMPS-Na-co-St/magnetite was mainly in the core of the composite magnetic particles. PAMPS-Na-co-St/magnetite particles display excellent inhibition of steel corrosion in 1 M HCl solution and can be considered as suitable materials for corrosion inhibition of steel in acidic chloride-containing environments. Polarization measurements indicate that a PAMPS-Na-co-St/magnetite particle acts as a mixed type-inhibitor and inhibition efficiency increases with inhibitor concentration. EIS spectra exhibit one capacitive loop, which indicates that the corrosion reaction is controlled by a charge transfer process.

## References

[1] Gao G., Wu H., He R., Cui D. (2010). Corrosion inhibition during synthesis of Cu_2_O nanoparticles by 1,3-diaminopropylene in solution. Corros. Sci..

[2] Zubillaga O., Cano F.J., Azkarate I., Molchan I.S., Thompson G.E., Cabral A.M., Morais P.J. (2008). Corrosion performance of anodic films containing polyaniline and TiO_2_ nanoparticles on AA3105 aluminium alloy. Surf. Coat. Tech..

[3] Behzadnasab M., Mirabedini S.M., Kabiri K., Jamali S. (2011). Corrosion performance of epoxy coatings containing silane treated ZrO_2_ nanoparticles on mild steel in 3.5% NaCl solution. Corros. Sci..

[4] Xu L., Chen X., Wu Y., Chen C., Li W., Pan W., Wang Y. (2006). Solution-phase synthesis of single-crystal hollow Cu_2_O spheres with nanoholes. Nanotechnology.

[5] Gurunathan K., Trivedi D.C. (2000). Comparison between two combustion routes for the synthesis of nanocrystalline SnO_2_ powders. Mater. Lett..

[6] Lenz D.M., Ferreira C.A., Delamar M. (2002). Distribution analysis of TiO_2_ and commercial zinc phosphate in polypyrrole matrix by XPS. Synth. Met..

[7] Liu Y., Huang J., Tsai C., Chuang T.C., Wang C. (2004). Changing the shape of ZnO nanostructures by controlling Zn vapor release: From tetrapod to bone-like nanorods. Chem. Phys. Lett..

[8] Lenz D.M., Delamar M., Ferreira C.A. (2003). Investigation of layered LiNi_1/3_Co_1/3_Mn_1/3_O_2_ cathode of lithium ion battery by electrochemical impedance spectroscopy. J. Electroanal. Chem..

[9] Ray S.S., Biswas M. (2000). Water-dispersible conducting nanocomposites of polyaniline and poly(*N*-vinylcarbazole) with nanodimensional zirconium dioxide. Synth. Met..

[10] Bhattacharya A., Ganguly K.M., De A., Sarkar S. (1996). A new Conducting nanocomposite-Ppy-Zirconium(IV) oxide. Mater. Res. Bull..

[11] Montoya P., Jaramillo F., Calderon J., de Torresi S.I.C., Torresi R.M. (2010). Evidence of redox interactions between polypyrrole and Fe_3_O_4_ in polypyrrole-Fe_3_O_4_ composite films. Electrochim. Acta.

[12] Montoya P., Martins C.R., de Melo H.G., Aoki I.V., Jaramillo F., Calderón J.A. Synthesis of polypyrrole-magnetite/silane coatings on steel and assessment of anticorrosive properties. http://www.sciencedirect.com/science/article/pii/S0013468613013741.

[13] Benda P., Kalendová A. (2013). Anticorrosionproperties of pigments based on ferrite coated zinc particles. Phys. Procedia.

[14] Boinovicha L.B., Gnedenkov S.V., Alpysbaeva D.A., Egorkin V.S., Emelyanenko A.M., Sinebryukhov S.L., Zaretskaya A.K. (2012). Corrosion resistance of composite coatings on low-carbon steel containing hydrophobic and superhydrophobic layers in combination with oxide sublayers. Corros. Sci..

[15] Wang P., Lee C., Young C.T. (2005). Preparation and clinical application of immunomagnetic latex. J. Polym. Sci..

[16] Hong R.Y., Pan T., Li H.Z. (2006). Microwave synthesis of magnetic Fe_3_O_4_ nanoparticles used as a precursor of nanocomposites and ferrofluids. J. Magn. Magn. Mater..

[17] Yu L.Q., Zheng L.J., Yang J.X. (2000). Study of preparation and properties on magnetization and stability for ferromagnetic fluids. Mater. Chem. Phys..

[18] Shchukin D.G., Radtchenko I.L., Sukhorukov G.B. (2003). Micron-scale hollow polyelectrolyte capsules with nanosized magnetic Fe_3_O_4_ inside. Mater. Lett..

[19] Butter K., Philipse A.P., Vroege G.J. (2002). Synthesis of properties of iron ferrofluids. J. Magn. Magn. Mater..

[20] Xu Z., Shen C., Tian Y., Shi X., Gao H.-J. (2010). Organic phase synthesis of monodisperseiron oxidenanocrystalsusingiron chlorideas precursor. Nanoscale.

[21] Atta A.M., El-Azabawy O.E., Ismail H.S., Hegazy M.A. (2011). Novel dispersed magnetite core-shell nanogel polymers as corrosion inhibitors for carbon steel in acidic medium. Corros. Sci..

[22] EL-Mahdy G.A., Atta A.M., Dyab A.K.F., Al-Lohedan H.A. (2013). Protection of petroleum pipeline carbon steel alloys with new modified core-shell magnetite nanogel against corrosion in acidic medium. J. Chem..

[23] Lu S., Ramos J., Forcada J. (2007). Self-stabilized magnetic polymeric composite nanoparticles by emulsifier-free emulsion polymerization. Langmuir.

[24] Ma M., Zhang Y., Yu W., Shen H., Zhang H., Gu N. (2003). Preparation and characterization of magnetite nanoparticles coated by amino silane. Colloids Surf. APhysicochem. Eng. Asp..

[25] Dubois L.H., Zegarski B.R., Nuzzo R.G. (1986). Spontaneous organization of carboxylic acid monolayer films in ultrahigh vacuum. Kinetic constraints to assembly via gas-phase adsorption. Langmuir.

[26] Unzueta E., Forcada J. (1995). Semicontinuous emulsion copolymerization of methyl methacrylate and n-butyl acrylate: 1. Effect of mixed emulsifiers in seeded polymerization. Polymer.

[27] Dyab A.K.F., Atta A.M. (2013). Microgel-stabilised non-aqueous emulsions. RSC Adv..

[28] Atta A.M. (2013). Synthesis and surface active amphiphilic 2-acylamido-2-methylpropane sulfonic acid-co-*N*-isopropyl acylamide nanoparticles in aqueous media. Int. J. Electrochem. Sci..

[29] Atta A.M., Dyab A.K.F., Allohedan H.A. (2013). A novel route to prepare highly surface active nanogel particles based on nonaqueous emulsion polymerization. Poly. Adv. Tech..

[30] Lu S., Forcada J. (2006). Preparation and characterization of magnetic polymeric composite particles by miniemulsion polymerization. J. Polym. Sci..

[31] Rosen M.J. (1985). Surfactants and Interfacial Phenomena.

[32] Asefi D., Arami M., Sarabi A.A., Mahmoodi N.M. (2009). The chain length influence of cationic surfactant and role of nonionic co-surfactants on controlling the corrosion rate of steel in acidic media. Corros. Sci..

[33] Negm N.A., Al Sabagh A.M., Migahed M.A., Abdel Bary H.M., El Din H.M. (2010). Effectiveness of some diquaternary ammonium surfactants as corrosion inhibitors for carbon steel in 0.5 M HCl solution. Corros. Sci..

[34] Lawrence D.B., Cai T., Hu Z., Marquez M., Dinsmore A.D. (2006). Temperature-responsive semipermeable capsules composed of colloidal microgel spheres. Langmuir.

[35] Brugger B., Rosen B.A., Richtering W. (2008). Microgels as stimuli-responsive stabilizers for emulsions. Langmuir.

[36] Sant M.M., Wang S., Laurent B., Sen S. (2011). Superparamagnetic iron oxide nanoparticles (SPIONs): Development, surface modification and applications in chemotherapy. Adv. Drug Deliv. Rev..

[37] Teo Y.Y., Misran M., Low K.H., Zain S.M. (2011). Effect of unsaturation on the stability of C18 polyunsaturated fatty acids vesicles suspension in aqueous solution. Bull. Korean Chem. Soc..

[38] Amin M.A., Khaled K.F., Mohsen Q., Arida H.A. (2010). A study of the inhibition of iron corrosion in HCl solutions by some amino acids. Corros. Sci..

[39] Bereket G., Yurt A. (2001). The inhibition effect of amino acids and hydroxy carboxylic acids on pitting corrosion of aluminum alloy 7075. Corros. Sci..

[40] Larabi L., Harek Y., Traisnel M., Mansri A. (2004). Synergistic Influence of poly(4-vinylpyridine) and potassium iodide on inhibition of corrosion of mild steel in 1 M HCl. J. Appl. Electrochem..

[41] Lebrini M., Lagrenee M., Vezin H., Traisnel M., Bentiss F. (2007). Experimental and theoretical study for corrosion inhibition of mild steel in normal hydrochloric acid solution by some new macrocyclic polyether compounds. Corros. Sci..

[42] Macdonald R., Franceschetti D.R., Macdonald J.R. (1987). Impedance Spectroscopy.

[43] Ma H., Chen S., Niu L., Zhao S., Li S., Li D. (2002). Inhibition of copper corrosion by several Schiff bases in aerated halide solutions. J. Appl. Electrochem..

